# Limits of functional illiteracy in explaining human misinformation: the knowledge illusion, values, and the dual process theory of thought

**DOI:** 10.3389/fpsyg.2024.1381865

**Published:** 2024-04-08

**Authors:** Giorgio Gronchi, Axel Perini

**Affiliations:** Section of Psychology, Department of Neuroscience, Psychology, Drug Research and Child's Health, University of Florence, Florence, Italy

**Keywords:** functional illiteracy, misinformation, fake news, knowledge illusion, values, dual process theory of thought, fast thinking

## Introduction

In 1949, UNESCO (United Nations Educational, Scientific, and Cultural Organization) recognized the ability to read and write as a fundamental right (Bhola, 1995; Vágvölgyi et al., 2016). In the subsequent years, this recognition led to a necessity for a clear and operational definition to delineate between literate and illiterate individuals, as well as to identify different proficiency levels. In 1978, the UNESCO General Conference stated: A person is literate who can with understanding both read and write a short simple statement on his everyday life. A person is illiterate who cannot with understanding both read and write a short simple statement on his everyday life. A person is functionally literate who can engage in all those activities in which literacy is required for effective functioning of his group and community and also for enabling him to continue to use reading, writing, and calculation for his own and the community's development. A person is functionally illiterate who cannot engage in all those activities in which literacy is required for effective functioning of his group and community and also for enabling him to continue to use reading, writing, and calculation for his own and the community's development (UNESCO, 1978, p. 183).

More recently, the functional illiteracy concept has been extended to activities that characterize the contemporary society. For example, Bugaievska (2012) distinguished the following forms of functional literacy: general, computer literacy, foreign language proficiency, information and communicative literacy, household literacy, literacy of behavior in emergent situations, socio-political literacy. Voronovych (2019) included also legal and general professional literacy, environmental literacy, civic literacy (ability to assess political and economic situation and make appropriate decisions).

Functional illiteracy is a quite widespread concept in scientific literature. An exception (see Section 3) is the thinking literature within psychology. Aside from that, different disciplines have employed this construct: education (Spaull, 2013), economics (Van der Berg et al., 2011), computer science (Zollo and Quattrociocchi, 2018), medicine (Badarudeen and Sabharwal, 2010) psychology (Bulajić et al., 2019).

Specifically, functional illiteracy has been employed to explain the tendency to believe in fake news, conspiracy theories and the general spread of misinformation (Koppel and Langer, 2020; Moscadelli et al., 2020; Moro and Fioravanzi, 2022; McPhedran et al., 2023). Even EU funded projects mention this connection.^1^ Many articles in newspapers and on social media also attest to functional illiteracy for explaining human foolishness and gullibility. From this point of view, the Italian case is of particular interest. The media dissemination of the OECD (2019) PIAC survey results, which report to a high number of functional illiterates in Italy, has received particular resonance in public discourse. Journalists, pundits, and even politicians resort to the widespread prevalence of functional illiteracy to justify behaviors contrary to their own beliefs and expectations (voting behavior, tendency to believe in fake news, anti-scientific beliefs). The Italian Wikipedia page *Analfabetismo Funzionale*^2^ confirms this relationship (differently from the English version) citing the Treccani encyclopedia, a medical website and a mathematical blog.

Given the widespread use of functional illiteracy by international organizations as well as within public discourse and across several disciplines, in this opinion paper, we criticize the use of this concept in light of its measurement issues and the literature on human misinformation.

## A problematic construct

In 2016, Vágvölgyi et al. published a review paper on the definition of functional illiteracy, its assessment, and the associated differential diagnoses. They pointed out that the construct of functional illiteracy has never been clearly defined operationally. The estimated number of functional illiterates is classically based on the Programme for the International Assessment of Adult Competencies (PIAAC, the measure that is periodically taken in over 40 countries), the International Adult Literacy Survey (IALS) or the Adult Literacy and Life Skills Survey (ALL). However, these measures do not explicitly refer to functional illiteracy (OECD and Statistics Canada, 2000), outlined as the inability to use one's own reading, writing, and calculation skills for his/her own and the community's development. Indeed, the tasks proposed by these three measures do not imply functional illiteracy (OECD and Statistics Canada, 2000; Vágvölgyi et al., 2016). The point here is that functional illiteracy was never operationally defined in psychometrics terms (see Supplementary material, Section S1).

Also, literature about functional illiteracy proposed diverse definitions and diagnostic assessment standards. Beyond PIAAC, ALL and IALS, other studies employed years of schooling to measure functional illiteracy (Bhola, 1995; Martinez and Fernandez, 2010; Vágvölgyi et al., 2016) or developmental delay (Eme, 2011; Rüsseler et al., 2013). In other cases, functional illiteracy is potentially confounded with illiteracy *per se* (Thompkins and Binder, 2003). Lastly, there are studies that call their sample “functional illiterate” without any justification (Van Linden and Cremers, 2008; Kosmidis et al., 2011). Thus, the whole picture of functional illiteracy assessment is particularly problematic, potentially making any estimation unreliable.

## Human misinformation

In the last years, the issue of misinformation has been the subject of numerous psychological studies (Arechar et al., 2023). Despite the extensive existing literature, in this section we will focus on the nature of human knowledge and the variables that impact why we believe certain things (Ecker et al., 2022).

Starting from the study of Rozenblit and Keil (2002) many studies have testified that people overestimate their knowledge and understanding about objects, phenomena, and concepts of the world around us. For example, taking into account a common-use vehicle such as a bicycle, generally people think that they know how it works and how the different parts of it interact. However, Lawson (2006) observed that people fail to recognize the correct picture of a bicycle among other bikes in which the different parts were arranged in the wrong way. Remarkably even expert cyclists have the same problem.

Recent research conducted by Sloman et al. (Sloman and Fernbach, 2017; Hemmatian and Sloman, 2018; Rabb et al., 2019; Light et al., 2022) explain this tendency in terms of the inability to distinguish one's own knowledge respect to the knowledge of other people and information that we can obtain from the environment. In other words, individuals rely on the community they are part of to outsource their understanding of the world. In line with this view, Kahan (2013) underlines that our beliefs are not independent data pieces resulting from a rational evaluation of evidence. Rather, individual beliefs are intricately connected to other beliefs, shared cultural values, and our identities. Kahan (2017) calls “identity protective cognition” the tendency to unconsciously dismiss evidence that does not reflect the beliefs that predominate in their group.

A particularly relevant case is that of fake news and ideological arguments. Aside from what we have already mentioned, the tendency to believe to false information has been put in relation with the dual process theory of thought (Kahneman, 2011). Basically, this theory posits the existence of two distinct cognitive systems that shape human decision-making and reasoning. The first (System 1 or fast thinking) is characterized by intuitive, automatic processes driven by affect and gut reactions. This system operates quickly and efficiently, providing rapid responses to stimuli. The second (System 2 or slow thinking) is reflective and involve deliberate and analytical thinking. It requires more cognitive effort and, provided there are sufficient cognitive resources, it can inhibit responses generated by fast thinking.

According to the Motivated Reasoning hypothesis (Kahan, 2013), the disposition to engage in slow thinking boosts ideological motivated positions. In this view, the disposition to think analytically may lead to use deliberative thinking to justify beliefs that are aligned to significant affinity groups and to protect their ideological identity (Kahan, 2013; Beck, 2017).

However, there is also evidence supporting the opposite pattern. For example, Pennycook and Rand (2019) observed that slow thinking was associated with the rejection or disbelief in fake news articles that align with one's political views. Thus, individuals may succumb to fake news due to a lack of deliberative thinking (for example, the inability to override fast thinking initial reaction based on shallow, ideologically acceptable information).

Given such complexity and the several factors that may affect the tendency to believe to false information, it can be useful to refer to a synthesis from David Rand^3^ (see also Pennycook and Rand, 2021). Referring to the tendency to believe to news headlines, he distinguishes factors that increase (i) belief regardless of truth and (ii) belief in falsehoods.

With regard to the former, perceived accuracy is affected by repeatedly seeing a claim (even if is outlandish, contrary to our ideology, or in the case of slow thinking-tendency), the consistency with the individual's values and own's beliefs, and the degree of trust in the source of information.

Regarding the belief in falsehoods, Rand lists the lack of slow thinking involvement (regardless of ideology) and thus elements that promotes the use of fast thinking (such as distraction, haste, coherence with prior knowledge, emotions) and the lack of digital/media literacy.

## Discussion

Net of the measurement issue, the current definition of functional illiteracy pertains to the inability to effectively participate in the society in which a person lives, despite being literate. This is attributed to a deficiency in procedural skills or knowledge in specific areas (e.g., legal, scientific, etc.). So, explaining human misinformation with functional illiteracy means that by providing enough knowledge and skills to people, they would be less prone to believing in fake news or develop fewer anti-scientific attitudes and beliefs.^4^

As seen in Section 3, individuals are extremely ignorant and rely on the community of people around them, the environment, and the surrounding technology to act in a certain way. This implies that providing factual information does not necessarily mean that this type of knowledge will be used to make decisions. The same applies to the acquisition of new skills: even acquiring new abilities does not guarantee that they will be used, to the extent that they depend on the aforementioned variables. It is important to note, however, that the acquisition of mere information may play a role (Sirlin et al., 2021), at least in the tendency to avoid fake news. However, the overall picture is much more complex, considering the role of factors such as values, epistemic dependence on one's own community, different modalities of thought, and so on.

Furthermore, we must consider the fact that illiteracy was much more widespread a century ago. However, the naive idea of the centrality of functional illiteracy to explain today's misinformation problems often seems to suggest that, paradoxically, contemporary society has greater issues in terms of knowledge and the ability to act effectively in the world compared to the past. Nevertheless, we have no empirical evidence on this aspect.

Moreover, paradoxically, those who invoke functional illiteracy to explain current misinformation and human dumbness are themselves victims of their own values and fast thinking: they find a simple cause (those who don't think like me are ignorant and/or have problems acting effectively in the world, in brief they are functional illiterate), ignoring that from a scientific literature suggests a much more complex picture (see Supplementary material, Section S2).

The pervasiveness to appeal to functional illiteracy to explain human dumbness may suggest that individuals are naturally inclined to explain errors in terms of a knowledge deficit. Future research could investigate whether, in the face of human dumbness, there exists a sort of heuristic that leads to favoring ignorance-based explanations over other accounts.

In summary, in this opinion paper, our aim is to caution against the temptation to explain human misinformation through a construct that, when interpreted in terms of the acquisition of facts and skills, is at least incomplete. Instead, considering that there is no precise definition of this construct, it is not possible to determine how much it impacts on human misinformation (and more generally on human dumbness) in light of a composite scenario that involves many different factors.

## Author contributions

GG: Writing – review & editing, Writing – original draft. AP: Writing – review & editing, Writing – original draft.

## Footnotes

^1^https://pedal-consulting.eu/skill-to-boost-innovation-and-professional-fulfillment-in-a-sustainable-economy-skill-bill/ (accessed February 3, 2024).

^2^https://it.wikipedia.org/wiki/Analfabetismo_funzionale (accessed February 3, 2024).

^3^https://davidrand-cooperation.com/home (accessed February 3, 2024).

^4^Within science communication, this idea is known as the deficit model (Miller, 1983; Bodmer, 1985; Sloman and Fernbach, 2017; Light et al., 2022).
