# Supplementary material for: Limits of functional illiteracy in explaining human misinformation: the knowledge illusion, values, and the dual process theory of thought
Source: Front Psychol. 2024 Apr 8;15:1381865. doi: 10.3389/fpsyg.2024.1381865 (PMC11033400; doi:10.3389/fpsyg.2024.1381865)
Supplement: Supplementary file 1 [file Presentation_1.pdf]

S1. Measurement issues of functional illiteracy

In 1978, the UNESCO proposed these definitions: "A person is literate who can with understanding both read and write a short simple statement on his everyday life. A person is illiterate who cannot with understanding both read and write a short simple statement on his everyday life. A person is functionally literate who can engage in all those activities in which literacy is required for effective functioning of his group and community and also for enabling him to continue to use reading, writing, and calculation for his own and the community's development. A person is functionally illiterate who cannot engage in all those activities in which literacy is required for effective functioning of his group and community and also for enabling him to continue to use reading, writing, and calculation for his own and the community's development" (UNESCO, 1978, p. 183).

In spite of the widespread use of the concept of functional illiteracy in many disciplines and in the public discourse, scientific understanding of individuals labeled as functionally illiterate is restricted (Vágvölgyi et al., 2016). Indeed, research on the definition of the construct, evaluation, and differentiation from related numerical and linguistic difficulties is infrequent and subject to controversy (Vágvölgyi et al., 2016).

Different (and arguable) criteria have been employed to label individuals as functionally illiterate: years of schooling to (Bhola, 1995; Martinez and Fernandez, 2010; Vágvölgyi et al., 2016), developmental delay (Eme et al., 2010; Rüsseler et al., 2013), illiteracy per se (Thompkins and Binder, 2003); no explicit criteria (Van Linden and Cremers, 2008; Kosmidis et al., 2011).

Regarding the international actions promoted by UNESCO, the OECD, and the IEA (International Association for the Evaluation of Educational Achievement), three types of surveys have been progressively developed and employed: the International Adult Literacy Survey (IALS, administered between 1994 and 1998), the Adult Literacy and Life Skills Survey (ALL, administered between 2002 and 2006) and the Survey of Adult Skills (PIAAC, administered between 2012 and 2017, and more recently, 2021-2022)[1]. Table 1 reports the date of administration and the different types of tasks for each survey.

Survey	Date	Tasks
IALS	1994-1998	Prose literacy<br>Document literacy<br>Quantitative literacy
ALL	2002-2006	Prose literacy<br>Document literacy<br>Numeracy<br>Problem solving
PIAAC	2012-2017<br>2021-2022	Literacy (prose + document)<br>Numeracy<br>Problem solving in technology/rich environments

Table 1. Survey proposed by UNESCO, OECD and IEA, years of administration and types of tasks (adapted from Vágvölgyi et al., 2016).

The IALS, ALL, and PIAAC assessments incorporate tasks related to prose and document literacy aimed at comprehending and utilizing information across various text formats. While all three assessments evaluate arithmetic skills through quantitative literacy and numeracy tasks, problem-solving tasks are exclusively featured in the ALL and the PIAAC study. Nevertheless, these studies

[1] For an historical perspective see https://nces.ed.gov/surveys/piaac/history.asp [accessed March 21, 2024].

typically approach literacy from a theoretical standpoint (see OECD working papers number 34-37) and do not offer diagnostic guidance concerning the assessment of functional illiteracy.

As Vágvölgyi et al. (2016) wrote the primary interest of the international political actors that have promoted these surveys is large-scale assessment, not in individual diagnostics. For this reason, they lack psychometric test criteria (reliability, construct validity, criterion validity), which are demanded in standard individual diagnostic tests (see also Evans, 2014). Indeed, in the official documentation these pieces of information are missing (Maehler & Rammstedt, 2020; Vágvölgyi et al., 2016; see also the official library of OECD[2]).

With regard to the most recent survey, the PIAAC, Evans (2014) specifically delved into the issue of content and external validity. Content validity refers to how well a test covers all relevant aspects of the construct: for example, numeracy is defined on the basis of four dimensions (context: everyday/personal, work, society and community, further learning; response to mathematical task: identify/locate/access information, act on/use information, interpret/evaluate information; mathematical content: quantity and number, dimension and shape, pattern and relationships, data and chance; representations of information: text, table, graphs). Each item can be classified across these four dimensions, alongside with its estimated difficulty. However, these are broad definitions that may be not suitable within specific countries. For instance, the four categories of context may or may not accurately reflect the range of actual, distinct social activities or settings in which an individual respondent in a specific country might participate in their life (Evans, 2014). Furthermore, regarding external validity, results in terms of averages or percentages of correct responses obtained in samples are typically used for rankings. However, in many cases, the uncertainty stemming from the estimation of that parameter in the corresponding population has been disregarded (Evans, 2014).

Progressively, Item Response Theory (IRT) has been applied to PIAAC. Given that each respondent is administered with a subset of items from the total item pool, it is inappropriate to use statistics based on the number of correct responses. IRT is the ideal solution for overcome this limitation and it has been applied to in order to estimate a standardized score across different surveys. Then, the score is usually interpreted in terms of five general levels of literacy or numeracy to make it meaningful (OECD, 2013; see Yamamoto et al., 2013, 2018). However, it should be noted that survey's results are often interpreted by media or national institutions ignoring the IRT analysis and recurring to simple mean values or proportion of correct responses (Evans, 2014). As Evans (2014) observed, this aspect is not under the control of the organizations conducting the survey. Nevertheless, the dissemination of these distorted and poorly interpretable results also affects research articles covering various fields.

Summarizing, the assessment of functional illiteracy is characterized by several issues. The lack of a clear and shared operational definition entails that the criteria for defining a functional illiterate can encompass many different aspects, often unrelated with the UNESCO definition (from years of schooling to simple illiteracy). Even the official OECD surveys suffer from the lack of an operational definition of functional illiteracy and psychometric test criteria (validity and reliability analyses).

In any case, even if there were a psychometrically validated test to measure functional illiteracy reliably and validly as defined by the OECD, such a construct would still play a partial role in the issue of misinformation/human dumbness given the complexity of the variables involved (see Section 3 and Section S2).

---

[2] https://www.oecd-ilibrary.org/ (accessed March 21, 2024).

S2. The use of functional illiteracy construct across different disciplines

The construct of functional illiteracy has been employed differently depending on the contexts (academic/non-academic) and scientific disciplines.

Firstly, given the supranational origin of UNESCO and OECD surveys, in individual countries, media, journalists, politicians, and non-academic institutions have often used the survey results and the concept of functional illiteracy (often in a distorted and uncritical manner) to support specific policies and advance their political agendas (Evans, 2014; Vágvölgyi et al., 2016).

Within academics, there is often a marked division between studies in psychology focusing on thinking and reasoning (thus more centered on cognitive aspects) and the rest of the literature, spanning across various other scientific fields (such as education, medicine, economics, and computer science).

Thinking and reasoning studies focusing on misinformation (and related issues such as human dumbness, fake news, anti-scientific thinking, etc) typically have overlooked the construct of functional illiteracy. Presumably this is due to the measurement issues described in S1 and the non-academic origin of this construct. As described in Section 3, thinking and reasoning research have elucidated the numerous factors (e.g., the knowledge illusion phenomenon, values, the role of fast and slow thinking) that affect human misinformation. Explanations based on the complex role of those factors are typically contrasted with explanations in terms of the deficit model (Bodmer, 1985; Miller, 1983; Light et al., 2022; Sloman & Fernbach 2017). According to this perspective, anti-scientific thinking, human dumbness and misinformation are primarily due to a lack of information or skills. Therefore, merely informing or acquiring skills should be sufficient to reduce the tendency toward misinformation and anti-scientific thinking. Given the OCSE definition of functional illiteracy, in this paper we have sustained that the functional illiteracy construct is a form of the deficit model. So, although the literature on thinking and reasoning does not explicitly refer to the concept of functional illiteracy, it implicitly criticizes the explanation based on this concept using instead another label, namely the deficit model. It should be noted that recent research (Sirlin et al., 2021) have attributed a role also to the acquisition of mere information in the tendency to avoid fake news. In particular, Sirlin et al. (2021) found that digital literacy predicts one's ability to discern truth from falsehood when assessing headline accuracy. Nonetheless, digital literacy is not a good predictor of users' intentions to share accurate as opposed to false headlines. This result underlines the considerable complexity that characterizes the phenomena of misinformation and human dumbness. Reducing the phenomenon to mere lack of factual knowledge and skills is inaccurate. Simultaneously, the deficit in information (or, in other terms, the issue of functional illiteracy) plays a role alongside many other variables (mentioned in Section 3). For this reason, it is important to invest in psychometric research concerning the operational definition and measurement of functional illiteracy, which, as we have seen in Section S1, is limited. In particular, the development of a clear operational definition should take into account research on the deficit model within the literature on thinking and reasoning.

However, beyond thinking and reasoning research, the concept of functional illiteracy is recurrently used as the primary explanation for misinformation, human dumbness, anti-scientific thinking and fake news receptivity across different domains. For example, recent research includes the economic and social costs of functional illiteracy (Cree et al., 2023), functional illiteracy as a primarily predictor of health bad decisions (Braz et al., 2023; Levic et al., 2023; Woods, 2023), fake news susceptibility (Dey et al., 2023) or the spread and prevention of functional illiteracy at the educational level (Kindl and Lenhard, 2023; Naheed et al., 2024).

In our view, the widespread adoption of this construct is based on a variety of reasons. First, the authority of the source plays a crucial role: the construct of functional illiteracy is proposed by international organizations (OECD, UNESCO) to which implicit high authority is attributed. Closely associated with this, there is social proof: media, politicians, and national institutions systematically report OECD survey results, referring to the issue of functional illiteracy. So, the social world around researchers confirms the validity of the construct (in line with the community of knowledge hypothesis, see Section 3 and Sloman and Fernbach, 2017). Thus, they are induced to employ this concept uncritically without examining the cognitive literature. Currently, the production of scientific articles has reached much higher levels than in the past: so, it is no easy to stay updated on the publications that occur across various specific domains. It is therefore plausible that researchers working in other fields with very different research traditions (education, economics, health, etc) may not be familiar with the literature on thinking and reasoning. Lastly, we believe that the explanation in terms of knowledge/skill deficits is particularly widespread because it is natural. An hypothesis that we have proposed in this opinion paper (and which we hope will be empirically tested in future research) is that people (and therefore researchers as well) intuitively appeal to the explanation based on knowledge deficit when they have to account for human dumbness, anti-scientific thinking, misinformation, etc. In other words, the first explanation that comes to mind is that of ignorance, whereas the intervention of other variables (values, nature of knowledge, distinction between two types of thinking) is not obvious.

We hope that this opinion paper will succeed in raising awareness among researchers who are not directly involved in the psychology of thinking about the complexity underlying the phenomenon of human disinformation and the problematic nature of an explanation based on functional illiteracy.

References not listed in the main paper

Braz, P. R., Moreira, T. R., Ribeiro, A. Q., de Faria, L. R., da Costa Carbogim, F., de Araújo Püschel, V. A. et al. (2023). COVID-19 Infodemic and impacts on the mental health of older people: cross-sectional multicenter survey study. *JMIR aging*, 6(1), e42707.

Cree, A., Kay, A., and Steward, J. (2023). *The Economic and Social Cost of Illiteracy: A Snapshot of Illiteracy in a Global Context.* World Literacy Foundation, 1-18.

Dey, P., Mishra, A., Das, R., Sonkar, A., & Agarwal, S. (2023). A Study on Perception of Fake News Disseminated over Social media During Covid 19 Pandemic. *Int. J. Res. Pub. Rev.*, 4(7), 376-381.

Evans, J. (2014). New PIAAC results: Care is needed in reading reports of international surveys. *ALM Int. J.,* 9(1), 37-52.

Kindl, J. and Lenhard, W. (2023). A meta-analysis on the effectiveness of functional literacy interventions for adults. *Ed. Res. Rev.,* 100569.

Levic, M., Bogavac-Stanojevic, N., Lakic, D., and Krajnovic, D. (2023). Predictors of inadequate health literacy among patients with type 2 diabetes mellitus: Assessment with different self-reported instruments. *Int. J. Env. Res. Pub. Heal.*, 20(6), 5190.

Maehler, D. B. and Rammstedt, B. (2020) (Eds.*). Large-Scale Cognitive Assessment. Analyzing PIAAC Data*. Springer Cham.

Naheed, R., Farid, S., and Ahmad, S. (2024). Prevalence and Correlates of Functional Illiteracy at College Level. *Ed. Res. Inn.* 4(1), 115-128

OECD (2013). *The Survey of Adult Skills: Reader's Companion.* Paris: OECD Publishing.

Woods, D. (Ed.) (2023). *Communication for Doctors.* Boca Raton: CRC Press.

Yamamoto, K., Khorramdel, L., and Shin, H. J. (2018). Introducing multistage adaptive testing into international large-scale assessments designs using the example of PIAAC. *Psychol. Test. Assess. Model.* 60(3), 347-368.

Yamamoto, K., Khorramdel, L., and Von Davier, M. (2013). Scaling PIAAC cognitive data. *Technical report of the survey of adult skills (PIAAC),* 408-440.
